# Case report: Surgery combined with extracorporeal membrane oxygenation for a patient with type A aortic dissection complicated with myocardial infarction after percutaneous coronary intervention

**DOI:** 10.3389/fcvm.2023.1205373

**Published:** 2023-07-07

**Authors:** Junjian Yu, Wenbo Yu, Hui Zeng, Jianfeng Gao, Jianxian Xiong

**Affiliations:** ^1^ Department of Cardiac and Vascular Surgery, The First Affiliated Hospital of Gannan Medical University, Ganzhou, China; ^2^ The First Clinical Medical College of Gannan Medical University, Ganzhou, China; ^3^ Department of Thoracic and Cardiac Surgery, Ningdu County People's Hospital, Ganzhou, China

**Keywords:** case report, aortic dissection, acute myocardial infarction, percutaneous coronary intervention, SUN’s procedure, extracorporeal membrane oxygenation

## Abstract

**Background:**

Aortic dissection (AD) is a severe cardiovascular disease characterized by aortic rupture, aortic valve insufficiency, aortic branch lumen stenosis, and occlusion. Acute ST-segment elevation myocardial infarction may be the primary manifestation when aortic dissection affects the coronary artery, leading to delayed or missed diagnosis of aortic dissection, and preventing patients from receiving timely and comprehensive treatment. Simultaneous aortic repair and coronary artery bypass grafting surgery are controversial because of their high mortality rates. Personalized and optimal treatment plans for patients should be taken seriously based on their different conditions and treatment options.

**Case presentation:**

A 42-year-old man who experienced 1 h of persistent precordialgia was admitted to a local second-level hospital for emergency treatment. Electrocardiogram (ECG) showed evidence of ST-segment elevation, and myocardial enzyme levels were CK-MB 18.35 ng/ml and troponin 0.42 ng/ml. The patient was treated for acute myocardial infarction (AMI) and urgently sent to the interventional catheter room. Coronary angiography showed stenosis of the starting part of the right coronary artery trunk. Thus, stent implantation was performed, and the stenosis section recovered patency; however, postoperative precordialgia was not alleviated. Computed tomography angiography (CTA) revealed a type A AD. The patient was immediately transferred to a higher-level hospital, underwent emergency surgery with cardiopulmonary bypass (CPB) ascending aorta replacement, SUN's procedure (total arch replacement and stented elephant trunk implantation), and simultaneous implantation of extracorporeal membrane oxygenation (ECMO), and regained consciousness within intensive care unit care. ECMO was discontinued when hemodynamics stabilized. The patient ultimately recovered well and was discharged.

**Conclusion:**

This case demonstrated that precordialgia is not limited to myocardial infarction but may also be accompanied by aortic dissection. Percutaneous coronary intervention (PCI) can timely and effectively restore coronary artery perfusion, strive for the opportunity of aortic repair surgery, and can overcome pump failure caused by myocardial infarction, cardiopulmonary bypass, heart block time, and myocardial ischemia-reperfusion injury. Personalized treatment is crucial for patients with complex type A aortic dissection.

## Introduction

Acute type A AD is a severe cardiovascular disease that may present with aortic rupture, aortic branch lumen stenosis, or occlusion. Hypoperfusion of the aortic branches caused by dissection of the false lumen can occur in all branches from the coronary artery opening to the abdominal aortic bifurcation (1). If the coronary artery is affected by acute type A AD, AMI may be the primary manifestation, and AD may be diagnosed late or go undetected if not fully considered (2).

Therapeutic methods for patients with AD complicated by AMI remain controversial. If a patient undergoes a one-stage surgery, including aortic repair and coronary artery bypass grafting (CABG), the mortality rate is often extremely high because of a severe setback of cardiac function, including existing myocardial infarction, CPB, heart block time, and myocardial ischemia-reperfusion injury. Owing to the high mortality rate, some scholars believe that implementing PCI treatment first is beneficial for such patients because it allows them to undergo second-stage aortic repair surgery and can reduce the mortality rate (3–5).

The etiology of acute chest pain in patients is not only limited to AMI but also includes the possibility of AD leading to coronary artery involvement and AMI (6). Early diagnosis can provide comprehensive treatment guidance. Notably, myocardial infarction may lead to heart pump failure. If a patient needs to undergo CPB aortic repair surgery again, the myocardial damage will undoubtedly worsen, leading to the inability to detach from the CPB. ECMO should be an indispensable treatment to maintain effective circulation and allow time for myocardial recovery.

In this article, we report a rare case of acute chest pain in a patient who was initially diagnosed with AMI and underwent PCI; however, the diagnosis of AD was delayed. After AD was confirmed, emergency aortic repair surgery was performed, and ECMO was implanted owing to heart pump failure. Based on relevant treatment experiences and a comprehensive literature review, we aimed to elucidate the clinical diagnosis and treatment of this unique and complex combined disease.

## Case description

**Figure 1:**
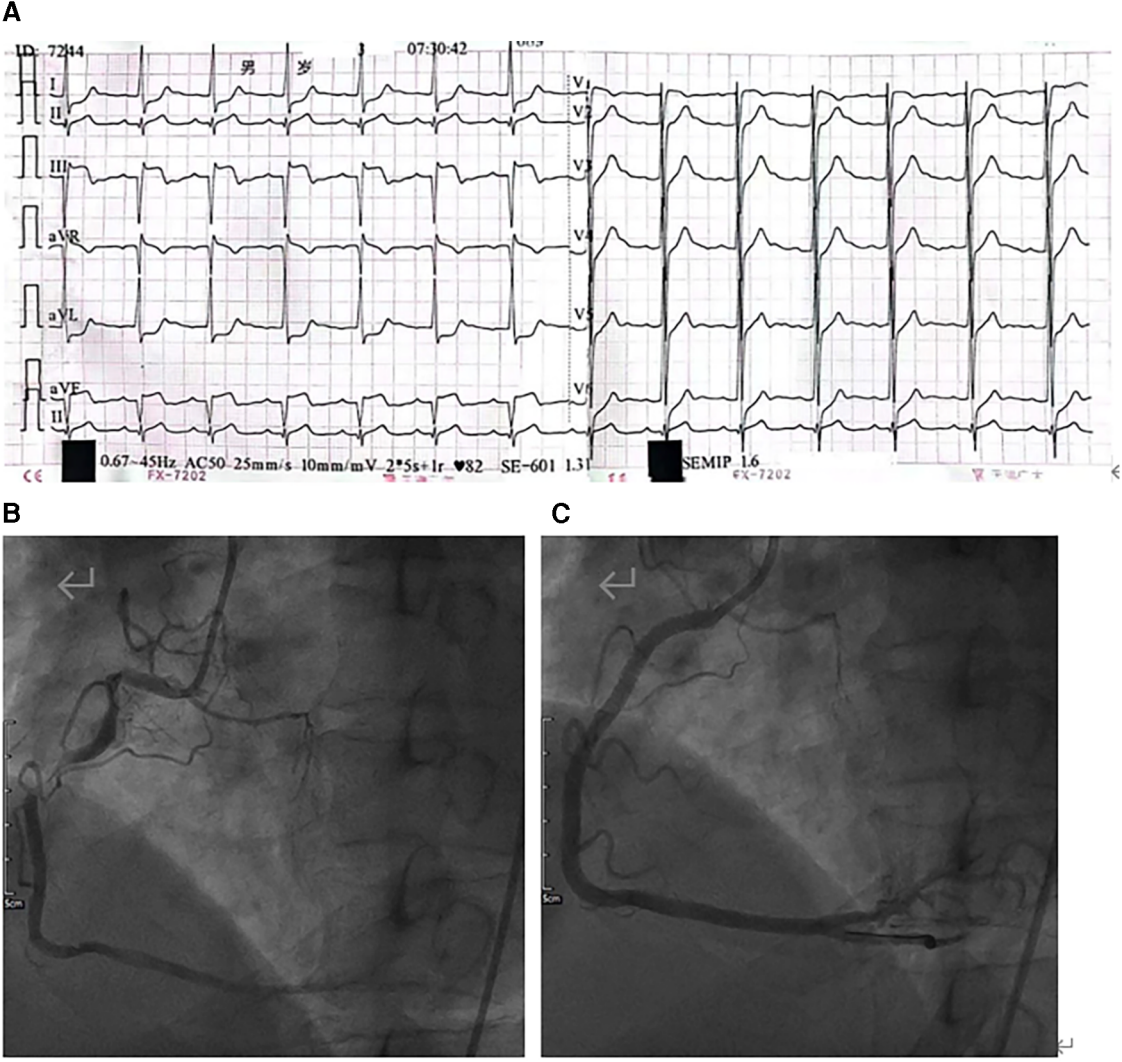
(**A**) Electrocardiogram (ECG) showing ST-segment elevation (II/III/aVF) and inferior acute myocardial infarction. (**B**) Coronary angiography showing irregular long-segment stenosis of the right coronary artery from the opening to the middle segment. (**C**) After stent implantation, the right coronary artery shows good morphology and an unobstructed vascular lumen.

**Figure 2:**
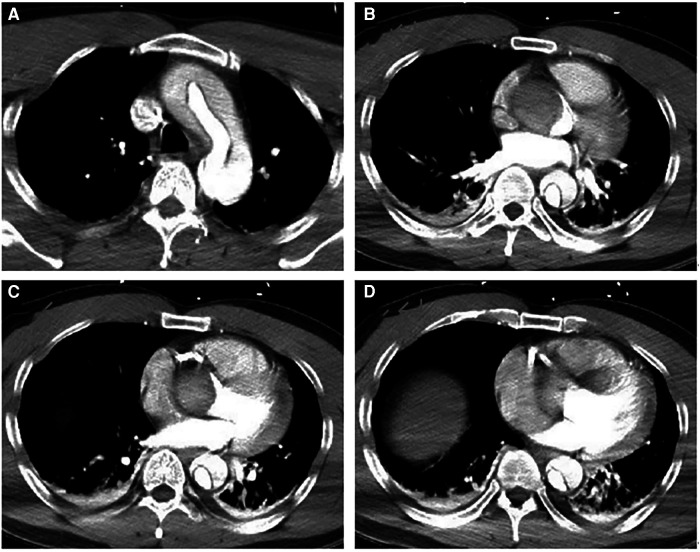
Preoperative aortic CTA image. (**A–D**) CTA shows the false lumen of the ascending aorta compressing the true lumen, extending above the opening of the right coronary artery. They indicate a thick hematoma in the false lumen compressing the right coronary artery.

**Figure 3:**
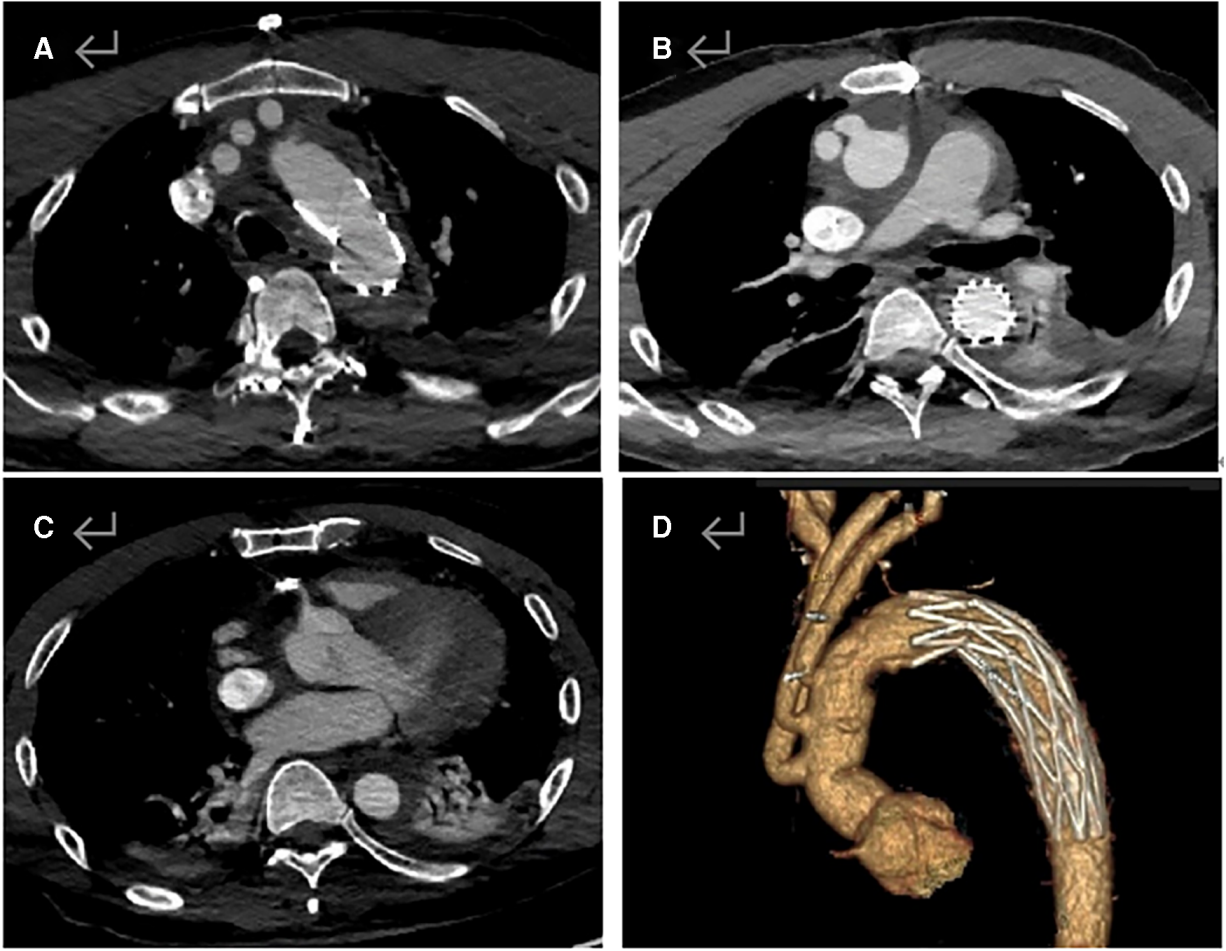
Postoperative aortic CTA image. (**A**) Artificial vessels in the aortic arch and the cephalobrachial artery are unobstructed. (**B**) Ascending aorta is unobstructed, and the stent is well dilated without internal leakage. (**C**) Opening of the right coronary artery is unobstructed, and stent implantation is visible. (**D**) Smooth morphology of the thoracic aorta.

**Figure 4:**
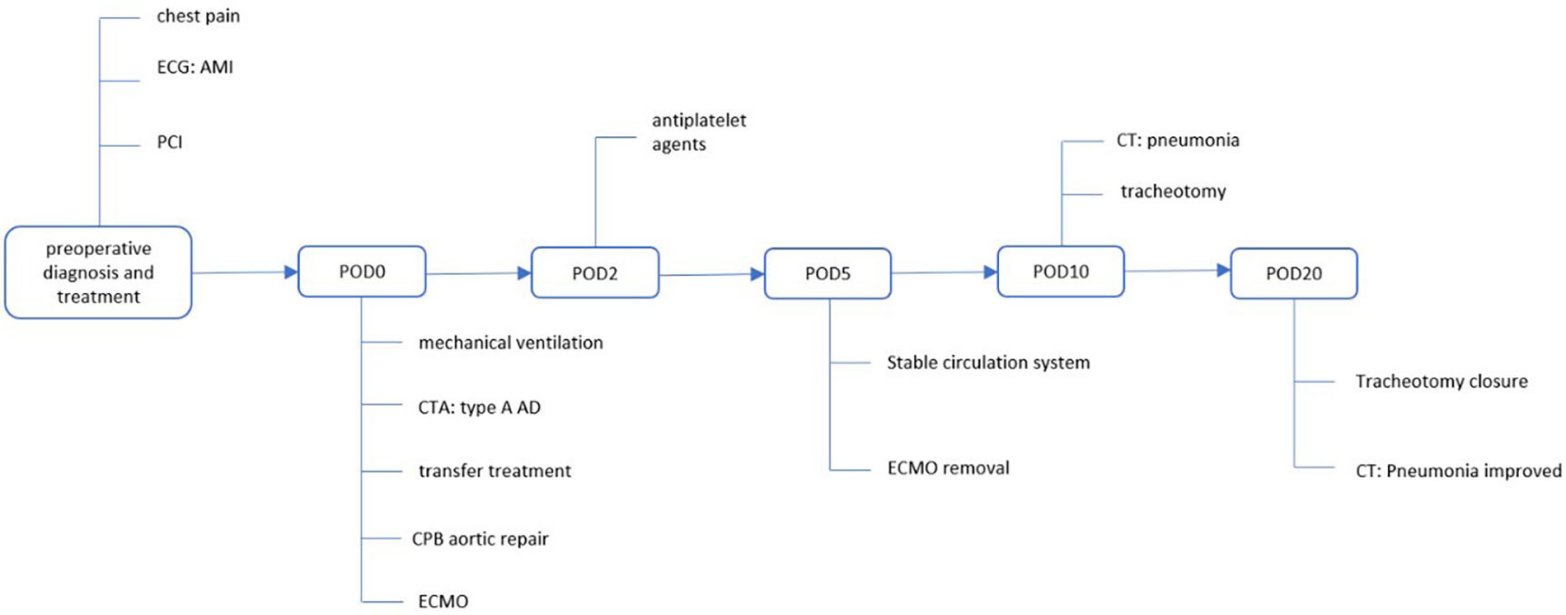
Timeline of the case.

A 42-year-old man (height 170 cm, weight 75 kg) who experienced severe precordialgia, which persisted for 1 h, was admitted to a local second-level hospital for emergency treatment. Although he had a history of hypertension for 5 years, he did not take antihypertensive drugs according to the advice of doctors. He had no trauma, surgery, or family history. There was no apparent cause of the sudden precordialgia. His blood pressure was 115/76 mmHg, and his heart rate was 105 bpm at admission. ECG showed ST-segment elevation (II/III/aVF) (Figure 1A), and myocardial enzyme levels were CK-MB 18.35 ng/ml and troponin 0.42 ng/ml. No scheduled cardiac ultrasound examination was performed, and the patient was treated for acute ST-segment elevation myocardial infarction, received 300 mg of aspirin and 180 mg of ticagrelor, and was immediately sent to the interventional catheter room. Coronary angiography showed severe stenosis of the starting part of the right coronary artery trunk (Figure 1B). Subsequently, PCI and coronary stent implantation were performed, as well as stenosis section recovery patency (Figure 1C). However, precordialgia was not alleviated after PCI, and no improvement was observed after the tirofiban injection. Moreover, hemodynamics became unstable, oxygenation function decreased, blood pressure dropped to 80/50 mmHg, peripheral blood oxygen saturation was 85%, and the heart rate was130 bpm, leading to mechanical ventilation 22 h after PCI and vasoactive drugs to maintain blood pressure. At this point, clinical physicians began to consider the possibility of AD, and an aortic CTA examination confirmed the diagnosis of type A AD (Figures 2A–D). The rupture of the aortic intima was located in the descending thoracic aorta, aortic dissection was reverse-tearing to the ascending aorta, a false lumen was extending to the upper edge of the right coronary artery opening, the hematoma inside the false lumen was very thick, and the true lumen of the ascending aorta became smaller due to compression by the false lumen. Local hospital staff urgently transferred the patient to a higher-level hospital, and emergency surgery was performed, including CPB (right axillary artery perfusion), ascending aorta replacement, and SUN's procedure (total arch replacement and stented elephant trunk implantation). It was difficult to detach the CPB during surgery because of right ventricular failure caused by preoperative myocardial ischemia. Therefore, VA-ECMO (left femoral artery and vein) was simultaneously implanted. The CPB time was 337 min, cardiac arrest time was 132 min, circulatory arrest time was 21 min, and intraoperative minimum temperature was 28°C. Owing to preoperative antiplatelet therapy, coagulation function had been severely affected, posing significant difficulties for surgical hemostasis. Platelets, cryoprecipitate, and fibrinogen were also infused. Fortunately, there was no severe progressive bleeding, and thoracic drainage on POD1 after the surgery was 500 ml. To prevent coronary stent thrombosis after PCI, platelet inhibitors on POD2 were re-administered. The patient regained consciousness on POD4 in the intensive care unit. Cardiac troponin and myocardial enzyme levels continued to decrease after surgery, circulation gradually stabilized, and the dosage of vasoactive drugs gradually decreased. ECMO was discontinued on POD5 when the hemodynamics returned to stability. Tracheotomy was performed on POD10 because of moderate pulmonary infection, and the postoperative mechanical ventilation time was 276 h. The tracheostomy was closed on POD20. Postoperative aortic CTA revealed that the artificial vessels were unobstructed (Figure 3A). The thoracic stent was well dilated without internal leakage (Figure 3B). The opening of the right coronary artery was unobstructed, the coronary stent implantation was visible (Figure 3C), and smooth morphology of the thoracic aorta was observed (Figures 3D, 4). The heart rate and blood pressure of the patient were stable, and heart function was normal through rehabilitation training after 6 months. Moreover, a cardiac ultrasound examination indicated normal cardiac structure and valve function, with a left ventricular ejection fraction of 55%.

## Discussion

Acute type A AD can be multifactorial and lead to rupture of the aortic intima and entry of circulating blood into the aortic wall, resulting in the formation of a false lumen. Acute type A AD is the most critical complication of cardiovascular surgery. The mortality rate of acute type A AD increases by 1% every hour within 48 h of onset. Conservative treatment has a high mortality rate of 50% within 2 days. Surgery is the preferred treatment for acute type A AD (7), whereas SUN's procedure is the standard procedure for Stanford type A AD (8).

The most common causes of death are dissection rupture, aortic valve insufficiency, ischemia of vital organs caused by aortic branch blood supply occlusion, and organ necrosis (9). The low perfusion of branch vessels is primarily due to the false lumen of the branch vessel intimal tear, which receives most of the blood; therefore, the false lumen expands and compresses the true lumen, resulting in insufficient blood supply to the organs. Acute type A AD can affect almost all aortic branches, from the coronary artery to the femoral artery. The incidence and severity of hypoperfusion differ among the various organs. Poor preoperative organ perfusion is a vital factor that significantly affects the prognosis of patients with acute type A AD (10). Accurate AD classification provides precise guidance on surgical treatment and evidence for disease prognosis (11). However, surgical treatment strategies are controversial when complex AD involves organs, such as the brain or other important branch arteries (12). In patients with AD complicated by AMI, preoperative myocardial ischemia, intraoperative cardiac blockade time, and ischemia-reperfusion injury have multiple impacts on cardiac function. The 5-year mortality rate in patients with AD and myocardial infarction is high, and surgical treatment can help reduce this mortality rate (13). However, concomitant CABG performed in acute type A AD repair surgery is associated with high in-hospital mortality (14–16).

The primary clinical manifestations of AD are persistent chest and back pain, and if complicated with AMI, precordium pain may persist. Acute type A AD is misdiagnosed or delayed because only acute coronary syndrome is considered when chest pain occurs. Therefore, the possibility of patients with AMI having AD should be considered (17–19). The acute coronary syndrome is not limited to simple coronary artery disease. AMI may also be the first manifestation of acute type A AD, which can be rapidly diagnosed through cardiac ultrasound and requires aortic CTA examination if necessary. Point-of-care ultrasound (POCUS) can quickly diagnose type A AD and change treatment strategies in patients with ST-segment elevation myocardial infarction (20). AD with concomitant AMI is prone to misdiagnosis, and transesophageal echocardiography (TTE) and D-dimer examination can help detect AD (21–23). Moreover, surveys have shown that for such patients, if a TTE examination is only performed after antithrombotic treatment, there is a correlation with a high surgery mortality rate (24). During the diagnosis and treatment of this case in a local secondary hospital, a cardiac ultrasound examination was overlooked. Therefore, we believe that even if a patient is diagnosed with simple AMI, a cardiac ultrasound examination should be performed before emergency intervention. In addition, aortic CTA can provide detailed information on the morphology of AD, including the location of intimal rupture and the involvement of branching vessels (25).

If AD diagnosis is missed, coronary angiography and PCI for patients with AMI are understandable, although interventional catheterization carries the risk of aortic rupture. However, if AD complicated by AMI can be accurately diagnosed simultaneously, PCI can be considered a priority treatment without urgent surgical procedures; some studies have been conducted accordingly (26, 27). The hospitalization mortality rate of patients with AD complicated by coronary artery involvement is as high as 40%. The first-stage PCI treatment strategy is of great help to patients undergoing aortic repair surgery and can reduce the mortality rate of such patients (28, 29). Cho et al. believed that using only guidewires to restore coronary artery perfusion could be an innovative interventional method as the first-stage treatment for coronary malperfusion caused by acute type A AD (30). In this case, although the diagnosis of AD was initially missed at a local secondary hospital, PCI did benefit the patient as it restored the right coronary artery supply and secured opportunities for referral and aortic repair surgery. However, it is equally difficult to determine the interval from PCI to aortic repair because patients with type A AD face the risk of aortic rupture at any time, even if first-stage PCI is successful. Therefore, we believe that aortic repair should be performed immediately after successful PCI. Similarly, not all types of coronary artery involvement caused by type A AD can be corrected using PCI. Some types can only be resolved through surgical repair of both the aorta and coronary arteries or through simultaneous CABG. Kreibich et al. recommended surgical strategies based on different coronary artery lesions, such as CABG for type C lesions and repair of coronary artery openings for type A and B lesions (31–33). Tong et al. reported that repair surgery is still possible even if the opening of the coronary artery intima is torn (34).

Due to the anatomical location of the coronary artery, right coronary artery involvement is more common than left coronary artery involvement in acute type A AD (35). Before undergoing AD center repair surgery, whether PCI is required in patients with concomitant right coronary artery hypoperfusion should be comprehensively evaluated, including concomitant cardiac tamponade, left and right ventricular function, and surgical team efficiency (36). Intraoperative coronary angiography is feasible if hospital conditions permit (37).

Intra-aortic balloon pump treatment is inappropriate because of aortic dissection etiology. ECMO should be used as an essential treatment for cardiac dysfunction to ensure that patients can be weaned from CPB and transferred to the intensive care unit (38, 39). Before VA-ECMO implantation, it is necessary to fully evaluate whether the artery to be intubated is affected by dissection, ensuring that the artery is supplied by the true lumen. Using the axillary artery for arterial catheterization ensures anterograde blood flow to the aorta (40–46). However, in this case, the right axillary artery was intubated for CPB, and the right femoral artery had just undergone a puncture for PCI. Fortunately, the left femoral artery of the patient was not affected by the dissection and was supplied by the true lumen of the aorta. Of course, it was also possible to consider intubating the branch of the aortic artificial vessel, which has the advantage of obtaining anterograde blood flow to the aorta. However, based on previous experience, in patients who had been treated with platelet inhibitors, if ECMO was established through branch intubation of artificial vessels, the impact of ECMO blood flow would lead to severe leakage of blood vessels. Therefore, left femoral artery catheterization was performed to establish ECMO. ECMO can significantly improve the survival rate of patients with cardiogenic shock caused by myocardial infarction or aortic repair surgery (47–53). With improvements in surgical technology and team efficiency, personalized and optimal treatment plans should be provided for patients based on their different conditions and treatment options.

## Conclusions

Our case demonstrated that caution regarding possible aortic dissection is required in patients with AMI. It also indicated that although PCI can serve as a transitional treatment if emergency aortic repair surgery cannot be performed, the aortic repair is fundamental, and ECMO must be prepared to overcome pump failure.

## Data Availability

The original contributions presented in the study are included in the article, further inquiries can be directed to the corresponding author.
